# Sonoselective transfection of cerebral vasculature without blood–brain barrier disruption

**DOI:** 10.1073/pnas.1914595117

**Published:** 2020-03-02

**Authors:** Catherine M. Gorick, Alexander S. Mathew, William J. Garrison, E. Andrew Thim, Delaney G. Fisher, Caitleen A. Copeland, Ji Song, Alexander L. Klibanov, G. Wilson Miller, Richard J. Price

**Affiliations:** ^a^Department of Biomedical Engineering, University of Virginia, Charlottesville, VA 22908;; ^b^Cardiovascular Division, Department of Medicine, University of Virginia, Charlottesville, VA 22908;; ^c^Department of Radiology & Medical Imaging, University of Virginia, Charlottesville, VA 22908

**Keywords:** focused ultrasound, microbubbles, endothelium, gene delivery

## Abstract

Treatment of many pathologies of the brain could be improved markedly by the development of noninvasive therapeutic approaches that elicit robust, endothelial cell-selective gene expression in specific brain regions that are targeted under MR image guidance. While focused ultrasound (FUS) in conjunction with gas-filled microbubbles (MBs) has emerged as a noninvasive modality for MR image-guided gene delivery to the brain, it has been used exclusively to transiently disrupt the blood–brain barrier (BBB), which may induce a sterile inflammation response. Here, we introduce an MR image-guided FUS method that elicits endothelial-selective transfection of the cerebral vasculature (i.e., “sonoselective” transfection), without opening the BBB. We first determined that activating circulating, cationic plasmid-bearing MBs with pulsed low-pressure (0.1 MPa) 1.1-MHz FUS facilitates sonoselective gene delivery to the endothelium without MRI-detectable disruption of the BBB. The degree of endothelial selectivity varied inversely with the FUS pressure, with higher pressures (i.e., 0.3-MPa and 0.4-MPa FUS) consistently inducing BBB opening and extravascular transfection. Bulk RNA sequencing analyses revealed that the sonoselective low-pressure regimen does not up-regulate inflammatory or immune responses. Single-cell RNA sequencing indicated that the transcriptome of sonoselectively transfected brain endothelium was unaffected by the treatment. The approach developed here permits targeted gene delivery to blood vessels and could be used to promote angiogenesis, release endothelial cell-secreted factors to stimulate nerve regrowth, or recruit neural stem cells.

## Significance

Focused ultrasound (FUS) is a targeted and noninvasive technique that can be used to activate gas-filled microbubbles (MBs) to oscillate within the bloodstream. This technique has been used previously to open the blood–brain barrier (BBB) to facilitate the delivery of therapeutics to the surrounding brain tissue. However, disruption of the BBB may be contraindicated in certain disease contexts. Here, we utilize low-pressure FUS to oscillate the MBs just enough to transfect endothelial cells, without opening the BBB. The low-pressure FUS regimen results in enhanced gene delivery to endothelial cells, with none of the inflammatory or immune pathway up-regulation observed at higher FUS pressures.

Pathologies of the brain, including neurodegenerative diseases, primary and metastatic brain tumors, cerebrovascular disease (stroke), and mental illnesses like depression and obsessive–compulsive disorder, are estimated to affect hundreds of millions of people worldwide (1–7). Gene therapy approaches for these diseases have shown promising preclinical results (8–13), but clinical treatment options for many of these conditions remain quite limited, due in large part to the difficulty of delivering therapeutics to the brain in a targeted manner. The blood–brain barrier (BBB), which includes a close network of tight junctions between endothelial cells to prevent paracellular diffusion, helps to isolate and protect the brain tissue from potentially harmful molecules in the systemic circulation but also prevents the uptake of many therapeutics from the bloodstream (14–17). Additionally, the skull presents a significant challenge for direct intracranial injections of therapeutics (18, 19), which are consequently very invasive and pose considerable surgical risks.

In light of these challenges to controlled delivery of therapeutics to the brain, focused ultrasound (FUS) has emerged as a promising approach to facilitate noninvasive, repeatable, and targeted drug and gene delivery to brain tissue across the BBB (20–24). Gas-filled microbubbles (MBs) can be introduced to the circulation intravenously. These MBs expand and contract in response to the acoustic pressure waves [which, at certain frequencies, can pass through bone without excessive attenuation (25)], pushing and pulling on endothelial cells to disrupt tight junctions, enhance transcellular transport (26–29), and induce transport of different molecules across the BBB (30–36). This method of using FUS in conjunction with MBs to transiently open the BBB has been used to deliver a wide range of therapeutic agents, including antibodies (37–39), proteins (40, 41), nanoparticles (21, 23, 42), and even stem cells (43, 44), to specific sites within the brain. The FUS modality has led to major breakthroughs for gene therapy for central nervous system pathologies as well (24, 45–48). Therapeutic agents can be coinjected into the bloodstream along with MBs or can be encapsulated within or linked to the MB shell to improve colocalization and enhance delivery (49–54). Widespread BBB disruption has even been shown to reduce amyloid-beta plaques in a mouse model of Alzheimer’s disease (55). The first clinical trials of FUS-mediated BBB disruption in human Alzheimer’s disease and glioma patients were recently completed, with no overt adverse effects (56, 57). Without question, FUS-mediated BBB disruption has proven to be an extremely valuable tool for noninvasive therapy for a wide range of cerebral pathologies.

Recent studies have revealed that, under certain conditions, opening of the BBB by FUS and MBs can induce an acute sterile inflammatory response in brain tissue (58–60). The inflammation induced by FUS has been shown to promote a wide range of beneficial effects, including immune activation and recognition of central nervous system tumors (61, 62), stimulation of neurogenic pathways that could permit regenerative therapies (58, 63, 64), and improving uptake of therapeutics from the bloodstream by increasing endocytosis and reducing small-molecule efflux (30, 65). However, disruption of the BBB may not be desirable in all cases where gene therapy has potential benefits. Following ischemic stroke, for example, the cerebral tissue is characterized by a high degree of instability and extensive acute and chronic inflammatory responses (66–69). In this scenario, further inflammation from BBB disruption, though transient and safe in many contexts, could pose a potential risk in the already-compromised microenvironment of the stroke ischemic penumbra. Other neurological conditions have also been associated with pathological inflammation (70), motivating the need for a gene therapy approach which avoids this potential FUS-induced sterile inflammatory response. Gene therapy targeted to endothelial cells could theoretically be utilized to permit modulation of the vasculature to promote angiogenesis, release endothelial cell-secreted factors to stimulate nerve regrowth, or recruit neural stem cells without affecting the BBB.

Endothelial cell sonoporation with FUS and MBs has been explored extensively in vitro. These studies have demonstrated the formation of membrane pores on endothelial cells following MB oscillation-induced shear stress as well as the initiation of intercellular gaps between adjacent cells and induction of endocytosis, all of which could facilitate the delivery of therapeutic agents (36, 71–78). The effects of acoustic sonoporation have been investigated in vivo as well (30, 31, 79), but to date no studies have utilized FUS to achieve targeted sonoporation of endothelial cells in vivo without disruption of tight junctions and/or enhancement of transcellular transport that would allow for therapeutic delivery beyond the vasculature.

In this study, we develop a method for endothelial-selective transfection of the cerebral vasculature without disruption of the BBB. We utilize low-pressure FUS to oscillate MBs such that we achieve endothelial cell membrane sonoporation without breaking tight junctions or enhancing transcellular transport and facilitating transport of the gene product beyond the blood vessels. This approach permits spatially targeted and cell-type-selective transfection in the brain without inducing inflammatory or immune responses.

## Results

### Peak-Negative Pressure of FUS Pulsing Can Be Modulated to Yield Sonoselective Transfection of Cerebrovascular Endothelium.

**Fig. 1.**
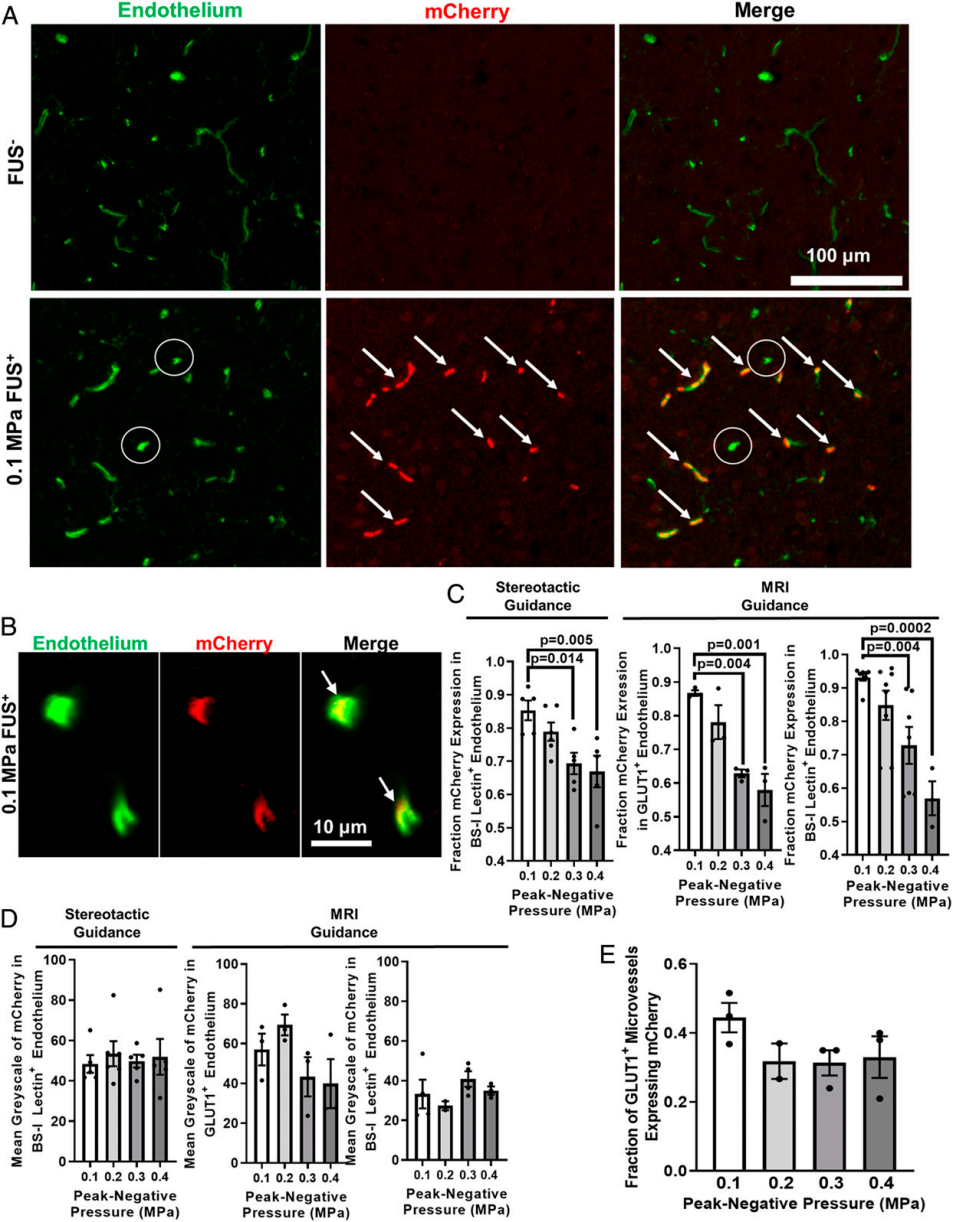
FUS peak-negative acoustic pressure (PNP) may be tuned to yield sonoselective cerebrovascular endothelial transfection. (*A* and *B*) Confocal images of FUS^+^ (0.1 MPa) and contralateral FUS^−^ brain tissue showing expression of mCherry reporter gene (red) with respect to endothelial cells (BS-I lectin, green). Arrows denote mCherry colocalization with endothelium. Circles denote untransfected capillaries. (*C*) Bar graphs of fraction of mCherry expression in cerebrovascular endothelium as a function of PNP. Highly selective endothelial transfection is observed at low PNPs (i.e., 0.1 MPa and 0.2 MPa). Similar relationships were observed when using both stereotactic and MR image guidance and both GLUT1 and BS-I lectin as endothelial markers. One-way ANOVAs followed by Dunnett’s multiple comparison tests. (*D*) Bar graphs of mean grayscale intensity of mCherry transgene expression in endothelium. Increasing PNP did not enhance endothelial mCherry fluorescence intensity. One-way ANOVAs followed by Dunnett’s multiple comparison tests. (*E*) Bar graph of fraction of GLUT1+ microvessels expressing mCherry. Increasing PNP did not increase the fraction of transfected microvessels. One-way ANOVA followed by Dunnett’s multiple comparison tests.

To test the hypothesis that reduced FUS peak-negative pressure (PNP) results in increased endothelial selectivity of transfection, we performed FUS-mediated gene delivery across a range of PNPs. Briefly, mCherry plasmid was first conjugated to cationic MBs without affecting MB size or stability. (*SI Appendix*, Fig. S1). MB–plasmid conjugates were delivered intravenously and the right striatum was targeted with FUS at PNPs ranging from 0.1 to 0.4 MPa (measured in water by hydrophone). Twenty-four hours later, the brains were harvested for staining to determine the overlap between mCherry expression and endothelial cells. The overlap between the endothelial markers (BS-I lectin or GLUT1) and mCherry expression was used to quantify the degrees of “endothelial-selective” transfection (mCherry signal that overlapped with one of the vascular markers) and “extravascular” transfection (mCherry signal which did not overlap with the vascular markers). In the area of the brain targeted with FUS, we observed robust mCherry expression (Fig. 1 *A* and *B* and *SI Appendix*, Fig. S2), while in the contralateral region of the brain (FUS^−^) little to no mCherry expression was detected (Fig. 1*A* and *SI Appendix*, Fig. S2). The fraction of “endothelial-selective” transfection ranged from 85 to 93% at the 0.1 MPa PNP and decreased with higher PNPs (Fig. 1*C*). This trend was consistent when both BS-I lectin and GLUT1 were used as endothelial markers, as well as when FUS was targeted under MRI guidance or with a stereotactic frame independent of image guidance. We have termed this phenomenon “sonoselectivity”—the ability to selectively transfect particular cell types by altering the ultrasonic parameters.

### Characterization of mCherry Expression in FUS-Transfected Cerebrovasculature.

After quantifying the endothelial selectivity of mCherry transgene delivery, we investigated additional metrics of mCherry transfection. In order to semiquantitatively assess the extent of transfection within the vasculature, the mean grayscale value of the mCherry staining within mCherry-positive vessels was compared across PNPs. There was no significant difference in this metric across PNPs under any of the conditions tested—MRI guidance versus stereotactic guidance, and BS-I lectin versus GLUT1 staining for endothelium (Fig. 1*D*). This indicates that there were no detectable changes in mCherry protein expression as a function of PNP. Additionally, there was no difference in the fraction of microvessels (as indicated by BS-I lectin or GLUT1 staining) positive for mCherry across PNPs (Fig. 1*E*). This finding is important for potential therapeutic applications of the sonoselective approach, as it demonstrates that the area of transfection coverage is not sacrificed for increased endothelial selectivity. Further investigation of the brains treated at 0.1 MPa was conducted to identify what type of vessels were being transfected. After extensive confocal microscopic examination of tissue sections from the 0.1-MPa group, we determined that mCherry transfection was confined to capillaries (Fig. 1*B*), with little to no evidence of mCherry expression in arterioles and venules (*SI Appendix*, Fig. S3). Finally, to assess whether transgene was expressed in off-target organs, we sonoselectively delivered a luciferase reporter plasmid with 0.1-MPa FUS to the cerebrovascular endothelium in a small cohort of mice (*n* = 3). Bioluminescence measurements showed that luciferase was indeed robustly expressed in FUS-targeted brains but was undetectable in off-target organs (i.e., heart, lungs, liver, and kidney) (*SI Appendix*, Fig. S4).

### Sonoselective Transfection of Cerebrovascular Endothelium Is Not Accompanied by Detectable BBB Opening.

**Fig. 2.**
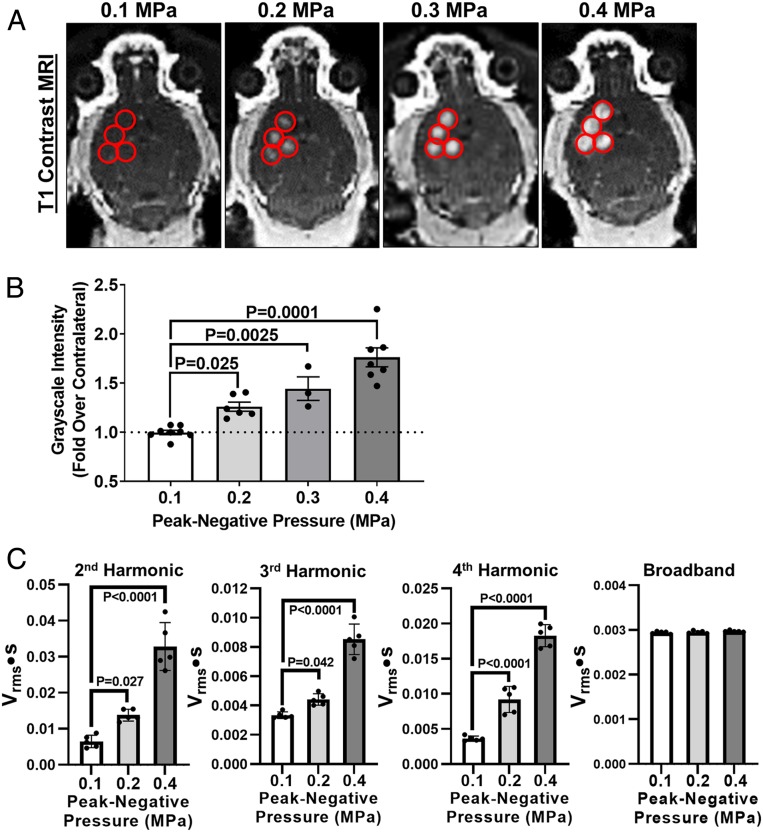
Sonoselective transfection of cerebrovascular endothelium is achieved without detectable BBB opening. (*A*) T1 contrast MR images of mouse brains after application of pulsed FUS in the presence of systemically administered MBs. FUS was applied at peak-negative PNPs ranging from 0.1 to 0.4 MPa in a four-spot sonication pattern. Sonication sites are denoted with red circles. Contrast is not detectable in FUS^+^ sites at 0.1 MPa but becomes visible at higher PNPs, indicating BBB opening. (*B*) Bar graph of contrast enhancement over contralateral FUS^−^ control hemisphere as a function of PNP. One-way ANOVA followed by Dunnett’s multiple comparison tests. (*C*) Passive cavitation analyses for second, third, and fourth harmonics, as well as broadband emissions. One-way ANOVAs followed by Dunnett’s multiple comparison tests.

T1-weighted MR images were collected before and after FUS, using a three-dimensional (3D) fast gradient echo pulse sequence, to guide FUS targeting (i.e., four spot sonication pattern in right striatum) and visualize contrast agent extravasation into brain tissue due to BBB disruption (Fig. 2*A*). At 0.1 MPa, there was no enhancement in signal intensity in the FUS-targeted regions, indicating a lack of BBB disruption. At increasing PNPs, we began to observe significant increases in the degree of signal enhancement and BBB opening (Fig. 2*B*). These results demonstrate that the sonoselective endothelial transfection at 0.1 MPa can be achieved independent of detectable BBB disruption. To assess MB activation as a function of PNP, acoustic emissions were recorded and analyzed after each treatment. Acoustic emissions at the second, third, and fourth harmonics increased significantly in concert with increasing PNP; however, no differences in broadband emissions were detected (Fig. 2*C*). Within the “sonoselective” 0.1-MPa group, second, third, and fourth harmonic emissions were remarkably consistent, showing little variability from treatment to treatment.

### Sonoselective Endothelial Transfection Is Not Associated with Significant Inflammatory or Immune Responses.

**Fig. 3.**
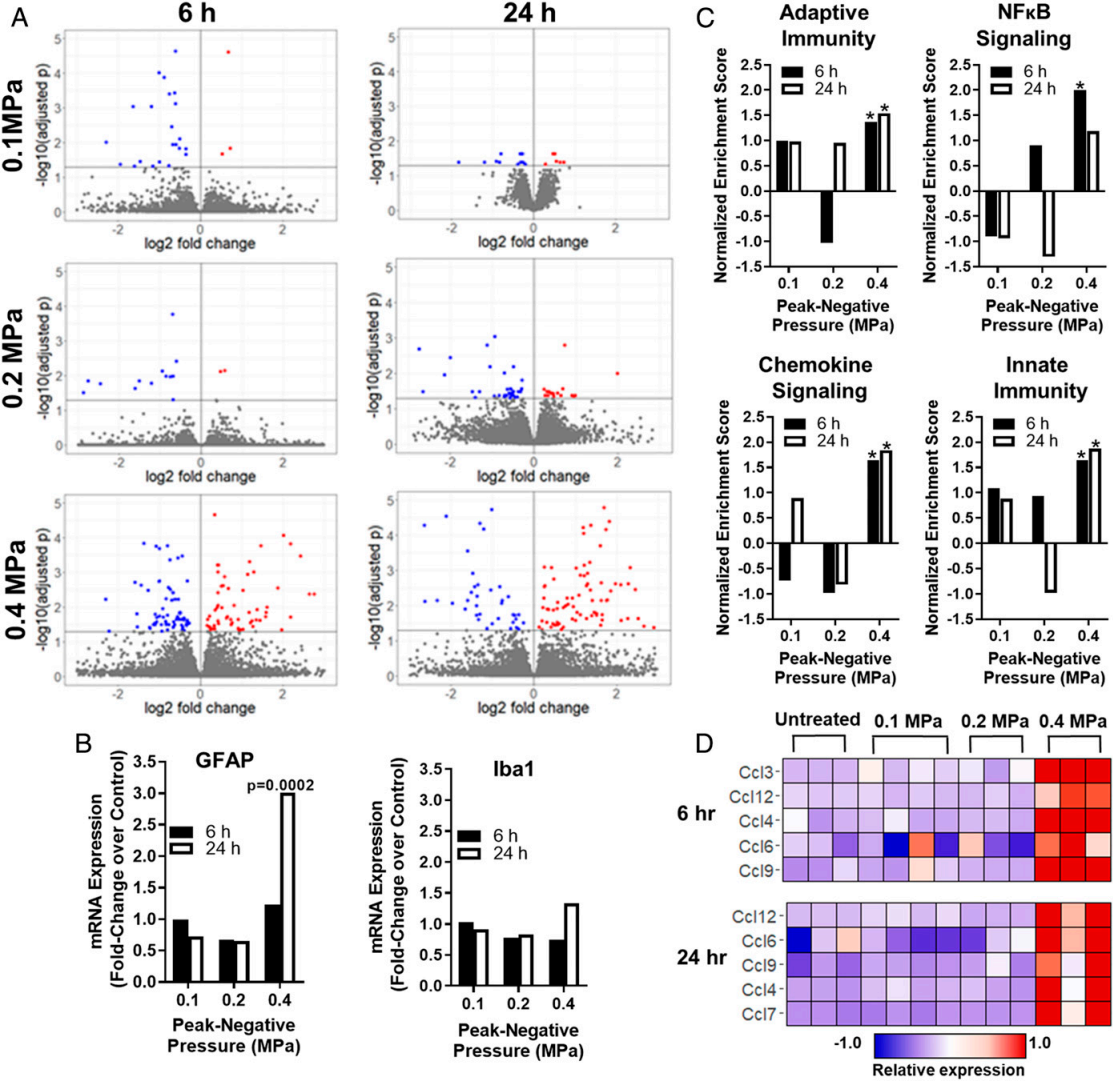
Sonoselective transfection of cerebrovascular endothelium at low PNP is achieved without eliciting a sterile inflammation response. (*A*) Volcano plots of differentially regulated transcripts at 6 and 24 h after pulsed FUS application to the brain (0.1 MPa [blue], 0.2 MPa [green], and 0.4 MPa [red]) in the presence of systemically administered MBs. Note the increase in differentially regulated transcripts with increasing NPN. (*B*) Bar graphs of transcripts used to assess astrogliosis (GFAP) and microgliosis (Iba1). Expression is shown as a fold change over normal brain tissue. No changes were observed in Iba1 expression, while GFAP expression was only increased at 24 h in response to 0.4-MPa FUS. (*C*) Gene-set enrichment analyses for selected pathways associated with inflammation and immunity. All pathways were significantly enriched at 0.4 MPa; however, none was enriched at 0.1 MPa or 0.2 MPa. *P < 0.05 vs. untreated brain tissue. (*D*) Expression levels of selected chemokines identified via leading-edge analysis of the “Chemokine Signaling” pathway. Each column corresponds to a single mouse.

To assess the impact of sonoselective transfection on brain tissue, we conducted a transcriptomic analysis of the FUS-treated brain tissue at 6 h and 24 h after FUS application. We investigated three PNPs for this analysis: 0.1 MPa, where we never observe detectable BBB disruption, 0.2 MPa, where we often see very minor BBB disruption, and 0.4 MPa, where there is routinely robust opening of the BBB and extensive contrast agent extravasation into the brain (Fig. 2). At either 6 or 24 h following FUS activation of plasmid-bearing MBs, the front right quadrant of the brain was harvested and processed for bulk messenger RNA sequencing followed by bioinformatics analyses (80). At both time points, we observed hundreds of differentially expressed genes at the 0.4-MPa PNP relative to naïve control animals, and far fewer at 0.2 and 0.1 MPa (Fig. 3*A*). We next investigated the differential regulation of key genes related to inflammatory and immune responses. Glial fibrillary acidic protein (GFAP), a marker of astrogliosis, was up-regulated at the 24-h time point in the 0.4-MPa group, but no up-regulation was observed in the 0.1- or 0.2-MPa groups (Fig. 3*B*). This is consistent with a greater potential for astrogliosis at the higher FUS PNPs that elicit detectable BBB opening, but not at lower FUS PNPs. Ionized calcium binding adaptor molecule 1 (Iba1), a marker of microgliosis, was not differentially expressed at any PNPs or time points, although it appears to be trending higher in the 0.4-MPa group at 24 h after FUS (Fig. 3*B*). Examination of several cytokine transcripts commonly associated with immunosuppression showed that they were neither significantly up-regulated nor down-regulated at any of the tested PNPs (*SI Appendix*, Table S1). It should also be noted that some markers of inflammation, including nuclear factor κB (NF-κB) pathway up-regulation, are largely resolved by 24 h, indicating that the sterile inflammation response is likely transient and reversible.

Gene-set enrichment analysis of the bulk RNA sequencing data revealed significant enrichment of numerous pathways associated with sterile inflammatory responses at the 0.4-MPa PNP level, including the reactome adaptive and innate immune system pathways, the chemokine signaling pathway, and the NF-κB pathway (Fig. 3*C*). Importantly, none of these pathways was enriched at 0.1 or 0.2 MPa (Fig. 3*C*). We then performed a leading-edge analysis of the chemokine signaling pathway gene set. Heat maps for the five most differentially expressed chemokines in this gene set at 6 h and 24 h for the 0.4-MPa group are shown in Fig. 3*D* and compared to the lower PNP groups. Chemokine expression was clearly and consistently higher in the 0.4-MPa group, wherein BBB opening was always evident.

We next examined the bulk RNA sequencing data to ascertain whether, independent of FUS application, systemically circulating cationic MBs could affect the brain transcriptome. In comparison to naïve brain tissue, we observed only minimal changes in gene expression at both 6 h and 24 h after cationic MB injection without FUS (*SI Appendix*, Fig. S5*A*). Furthermore, gene-set enrichment analysis of the RNA sequencing data indicated that no pathways associated with inflammation and/or immunological responses were significantly enriched or suppressed by cationic MBs alone (*SI Appendix*, Fig. S5*B*).

### Sonoselective Transfection Does Not Significantly Affect the Cerebrovascular Endothelial Transcriptome.

**Fig. 4.**
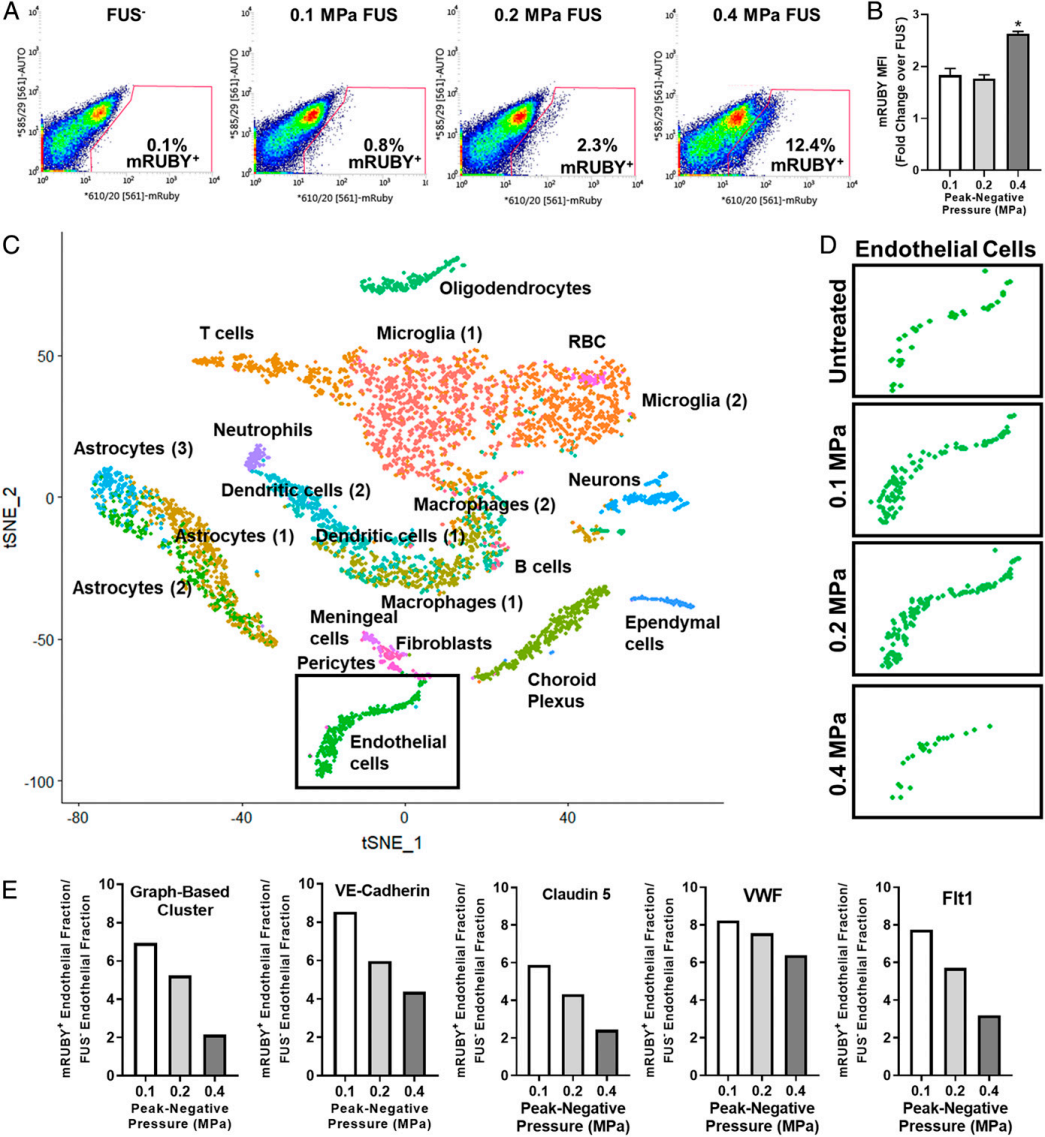
Cell identification and enrichment using flow cytometry and single-cell RNA sequencing. (*A*) Flow cytometry gating used to sort mRUBY^+^ transfected cells from whole-brain tissue samples. The mRUBY^+^ fraction increased with PNP. (*B*) mRUBY MFI for each PNP. *P < 0.0001 vs. 0.1 and 0.2 MPa. One-way ANOVA followed by Dunnett’s multiple comparison tests. (*C*) tSNE dimensionality reduction of aggregate sample containing cells from untreated and 0.1-MPa FUS, 0.2-MPa FUS, and 0.4-MPa FUS treatment groups. Labels on graph identify corresponding cell clusters. Endothelial cells (green) are boxed. (*D*) Endothelial cell clusters from tSNE analyses of sham (all cells, not sorted) and FUS-transfected (mRuby+ only) samples. Endothelial cell cluster size comparisons between FUS-treated groups reflect the relative proportion of transfected cells identified as “endothelial” at each PNP. (*E*) Bar graphs showing that low PNPs markedly enrich the endothelial fraction of transfected cells. The method used to identify endothelial cells (i.e., graph-based clustering or expression of individual markers of brain endothelium, such as VE-Cadherin, Claudin 5, Von Willebrand Factor [VWF], or Vascular Endothelial Growth Factor Receptor-1 [Flt1]) did not significantly affect the relationships between PNP and endothelial enrichment.

We then performed fluorescence-activated cell sorting (FACS) and single-cell RNA sequencing studies to both confirm that low PNP FUS markedly enriches the endothelial cell fraction of transfected cells and to determine whether sonoselective transfection alters the endothelial transcriptome. FACS was first used to isolate mRUBY^+^ cells from brain tissue wherein mRUBY plasmid-bearing MBs were activated with FUS at 0.1, 0.2, and 0.4 MPa. Brain tissue from sham mice that received mRUBY plasmid-MB injection, but without FUS application, comprised a sham control and were used to generate the flow cytometry gating scheme (Fig. 4 *A*, *Left*). The fraction of mRUBY^+^ cells isolated from the total population increased with FUS PNP (Fig. 4*A*), and the mean fluorescence intensity (MFI) of mRUBY was significantly enhanced at 0.4 MPa (Fig. 4*B*). Single-cell RNA sequencing was then performed on mRUBY^+^ cell populations from 0.1-, 0.2-, and 0.4-MPa–treated mice, as well as from sham mice [which received MBs but not FUS, and were not sorted for mRUBY^+^ (81)]. There were 2,000 cells sequenced from each treatment group. T-distributed scholastic neighbor embedding (tSNE) followed by graph-based clustering was used to group transcriptomally similar cell populations (Fig. 4*C*). Endothelial cell clusters were disaggregated based on treatment condition and reproduced in Fig. 4*D*. The proportion of FUS-transfected cells that were endothelial was enhanced and inversely related to PNP when compared to the baseline proportion of endothelial cells in sham, non-FUS-treated brains, confirming that the endothelial fraction of total transfected cells is enriched with low PNP FUS and diminished with high PNP FUS. At higher PNPs, the fraction of transfected cells which are endothelial is similar to the fraction of endothelial cells present in the brain at baseline, indicating no particular transfection selectivity. At lower PNPs, a greater fraction of the transfected cells was endothelial, suggesting a greater degree of sonoselectivity at these PNPs. This result is quantified in Fig. 4*E*, which illustrates that the relationship between FUS PNP and the enrichment of the endothelial fraction of transfected cells is independent of the expression marker (VE cadherin, Claudin 5, Flt-1, or VWF) used to identify any given cell as “endothelial.” This is consistent with the BSI-lectin and GLUT-1 immunohistochemistry results in Fig. 1, which show an increased proportion of endothelial cells transfected at lower PNPs.

**Fig. 5.**
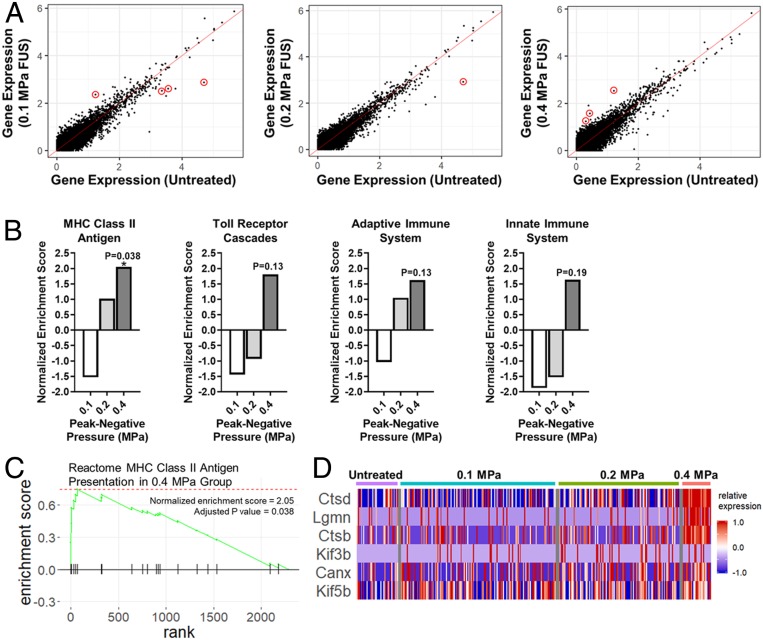
Single-cell RNA sequencing analyses indicate that sonoselective transfection with low-pressure FUS does not significantly affect the transcriptome of brain capillary endothelial cells in vivo. (*A*) Expression of individual genes in mRUBY^+^ endothelial cells transfected with 0.1-, 0.2-, and 0.4-MPa FUS in comparison to the expression of the same genes in untreated endothelial cells. Only eight total transcripts were differentially expressed (*P* < 0.05; red circles). (*B*) Selected gene-set enrichment analyses for mRUBY^+^ endothelial cells. While some gene sets associated with inflammation approached significance in the 0.4-MPa group, only the MHC Class II Antigen Presentation gene set was significantly enriched compared to untreated endothelium. (*C*) Enrichment plot for the MHC Class II Antigen Presentation gene set. (*D*) Leading-edge analysis of the MHC Class II Antigen Presentation gene set, showing that the Ctsd, Lgmn, and Ctsb transcripts predominantly drive enrichment of this gene set at 0.4 MPa.

Finally, we analyzed the transcriptomes of all mRUBY^+^ endothelial cells via single-cell RNA sequencing and compared them to untreated brain endothelium. In general, the transfected endothelium was remarkably quiescent. In total, only eight transcripts were differentially expressed among all three FUS PNPs (Fig. 5*A*). Gene-set enrichment analysis revealed that, for the 0.1- and 0.2-MPa groups, no gene sets were significantly enriched or repressed. For the 0.4-MPa group, Major Histocompatibility Complex (MHC) Class II Antigen Presentation was the only significantly enriched gene set (Fig. 5*B*). The Toll Receptor Cascades and Adaptive Immune System gene sets were only significant at *P* = 0.13, while the Innate Immune System gene set was only significant at *P* = 0.19 (Fig. 5*B*). Running enrichment score and leading-edge analyses for the MHC Class II Antigen Presentation gene set at 0.4 MPa are shown in Fig. 5 *C* and *D*, respectively. In the leading-edge analysis, each column corresponds to an individual endothelial cell. Enrichment of the MHC Class II Antigen Presentation gene set was driven by Ctsd, Lgmn, and Ctsb, which clearly exhibit enhanced expression at 0.4 MPa when compared to the other three groups. Due to the high cost of single-cell RNA sequencing, Figs. 4 and 5 represent the findings from a single trial in which three brains from each treatment condition were pooled for each sample.

## Discussion

Clinical outcomes for many brain pathologies could benefit appreciably by the introduction of new MR image-guided and noninvasive gene therapies that specifically modulate the function of the endothelial cell component of the BBB. In recent years, MB activation with FUS has been advanced as a mechanism for targeted gene delivery to the brain, albeit exclusively as a tool to disrupt the BBB and facilitate the transfection of brain cells that physically reside beyond the cerebral vasculature (e.g., neurons, astrocytes, and microglia). Here, we demonstrate that FUS PNP can be modulated to achieve so-called sonoselective (i.e., ∼90% cell specificity) endothelial transfection without the use of a cell-specific promoter. Of note, FUS application in this regime was accompanied by clearly demarcated and remarkably consistent acoustic harmonic emissions signatures that we propose could be exploited to eventually control sonoselective endothelial treatments in future applications. Bulk RNA sequencing confirmed that sonoselective endothelial transfection was achieved without eliciting a sterile inflammation response, while single-cell RNA sequencing indicated that the transcriptome of sonoselectively transfected endothelium was unaffected by treatment. Because BBB integrity is preserved, this noninvasive platform approach for cerebrovascular endothelial gene therapy may be especially powerful for conditions wherein even transient BBB disruption might pose a significant risk.

### Sonoselective Transfection of Endothelium without Use of Endothelial-Specific Promoters.

The sonoselective transfection regime demonstrated here facilitates increased transfection of endothelial cells at low FUS PNPs. This finding is evidenced by both the increased overlap between a fluorescent transgene and markers of endothelium in immunofluorescent staining, as well as the increased population of endothelial cells among the transgene-positive population identified using FACS and single-cell RNA sequencing. Importantly, this selective transfection is achieved without the use of a cell-type-specific promoter. For endothelium, this aspect is especially significant as endothelial cell-specific promoters can yield weak and variable transgene expression across different tissue beds, and particularly in the brain (82–85). The ability to target transfection to the endothelium without a cell-specific promoter allows for greater flexibility in gene therapy design. We can take advantage of the increased persistence or magnitude of transfection provided by some constitutive promoters to achieve a larger effect in the targeted cells for the same plasmid dose, opening the door to many future applications for noninvasive alterations of regions of the cerebral vasculature.

In this study, we made use of the constitutively active cytomegalovirus promoter, which has a relatively short “lifespan,” with expression peaking at 24 to 48 h. We envision this sonoselective approach as a short-term therapy to initiate recovery after injury or disease; however, the system could also be adapted to utilize a viral delivery vector for permanent transgene expression. Alternatively, a longer-acting promoter could be used in conjunction with the nonviral system to permit extended but transient gene expression. A prior study by our group demonstrated that FUS-mediated transfection of the brain with a nonviral vector driven by the beta-actin promoter results in sustained gene expression for >4 wk (23).

### In Vivo Endothelial Cell Sonoporation without Tight Junction Disruption or Transcellular Transport.

There are many mechanisms by which FUS and MBs stimulate cellular uptake of therapeutic agents (30, 86). The oscillation of MBs close to the plasma membrane of cells has been shown to push and pull on the membrane to cause membrane deformation and pore formation (87). The efficacy of membrane permeabilization has been shown to positively correlate with the oscillation amplitude of the MBs as well (73). Stable oscillation of the MBs results in local steady fluid flow around the bubbles, which is known as microstreaming. This microstreaming places shear stress on nearby cell membranes. This shear stress varies with the acoustic pressure driving the MB oscillation (88), and even MBs activated at low acoustic pressures (0.2 MPa) can generate enough stress to potentially damage vascular endothelium (89). We hypothesize that these physical mechanisms of membrane disruption are taking place across the range of FUS PNPs tested here (0.1 to 0.4 MPa). While previous studies have demonstrated that FUS and MBs can result in the disruption of endothelial tight junctions (27, 90), as well as increased transcellular uptake via vesicular transport (91, 92), these effects appear to be minimal with our 0.1-MPa treatment. We do not observe any contrast agent enhancement on T1-weighted MRI after 0.1-MPa FUS and MBs, and the transgene expression at this PNP is nearly all confined to the endothelium, suggesting that at this low PNP the MB oscillation is sufficient to sonoporate the endothelial cell membranes but not sufficient to disrupt tight junctions or promote additional transcellular transport.

### Harmonic Emissions Can Be Used to Monitor and Control MB Activity and Associated Bioeffects.

As the field of FUS-mediated therapeutic delivery to the brain gains momentum and moves closer to regulatory approval in human patients, there remains an ongoing concern over the potential dangers of neuroinflammation and petechiae due to damage caused by oscillating MBs. Additionally, variability in skull shape and thickness can result in variability in the bioeffects and treatment efficacy of FUS with MBs across and within test subjects (93–95). The concern over inflammation and treatment variability led to the development of monitoring systems to assess MB activity within the brain and the use of these observations to inform treatments. These systems utilize the principle of passive cavitation detection (PCD). PCD involves recording the acoustic emissions produced by the oscillating MBs within the skull. These emissions can then be correlated with the biological effects of different FUS exposures and used to monitor and control treatments to avoid unwanted adverse events (32, 96–98). There have been many recent advancements in cavitation monitoring to facilitate features like real-time control and feedback systems and enhanced 3D cavitation cloud mapping (99, 100), all with the intention of developing increasingly sensitive methods to ensure the safety of MB activity within the brain. In this study, we utilized PCD during FUS treatments and found that harmonic emissions increased significantly with increased FUS PNPs, with little to no broadband noise being detected (indicating that these treatments are occurring below the inertial cavitation threshold of the MBs). The large difference in harmonic emissions between 0.1 and 0.2 MPa, as well as the low degree of variability across animals at 0.1 MPa, suggests that emissions within this “sonoselective” regime are distinct and reproducible. This is ideal for ease of recognizing the “sonoselective” signature in future treatments and controlling FUS PNP to maintain bioeffects in this regime. Such control will be key for the long-term clinical applicability of FUS-activated MB technology in the brain.

### Sonoselective FUS Regime Does Not Induce a Sterile Inflammatory Response.

The finding that BBB opening by FUS and MBs can stimulate an acute sterile inflammatory response (58–60) has raised some concerns over the use of FUS and MBs for noninvasive therapeutic delivery in the brain in specific disease contexts. While the induction of a temporary inflammatory response can be justified for many disease applications, there are some contexts in which even a transient effect of this kind could induce more damage than the therapeutic delivery can justify. We wanted to design a treatment approach that could be used for these especially sensitive disease microenvironments, where any additional inflammation could have serious consequences. FUS activation of MBs at PNPs resulting in sonoselective endothelial transfection did not show any enrichment of genes or pathways related to adaptive or innate immune responses, or inflammatory signaling, demonstrating the potential of this approach for safe, noninvasive, targeted gene transfection in the cerebral vasculature in settings where BBB opening poses excessive risks.

### Sonoporation with Low-Pressure FUS Does Not Significantly Alter the Endothelial Cell Transcriptome.

In addition to avoiding systemic inflammation and immune activation, we wanted to ensure that our transfection approach would not cause significant damage to the transfected endothelium. After identifying the endothelial cell population from the transfected cells using single-cell RNA sequencing, we looked at the differentially regulated transcripts across the different FUS PNPs. Only a small number of individual genes were differentially regulated, at any of the FUS PNPs. The only gene set that displayed a significant difference was the MHC Class II Antigen Presentation gene set, which was up-regulated at 0.4 MPa, but not 0.2 or 0.1 MPa. A few other gene sets related to inflammation and immune response approached significant up-regulation at 0.4 MPa, but at 0.1 MPa the endothelium remained remarkably quiescent. This finding is promising for potential applications of this approach in pathologies which affect the cerebral vasculature. However, while the endothelium appears quiescent at 24 h after FUS, an important area for future investigation would be to assess the transcriptome at more acute time points. This would allow us to determine whether there is a transient response by endothelial cells which resolves by 1 d after treatment, or if the transfection does not in fact induce significant changes in gene regulation at any point.

### Potential Clinical Implications.

Given our results, low-PNP FUS with MBs could represent a therapeutic strategy for gene delivery to the cerebral vasculature for the treatment of a number of pathologies of the brain. By taking advantage of the phenomenon demonstrated here that endothelial membrane sonoporation is possible without extensive enhancement of transcellular transport and disruption of tight junctions (and thus, the BBB), we can deliver therapeutic genes to the vasculature even in sensitive disease settings. We envision using this platform to deliver proangiogenic and proarteriogenic genes to the vasculature in the context of ischemic stroke, as well as genes to stimulate the recruitment and differentiation of neural stem cells. Another application would be to transfect the endothelium with a gene for a transporter of some kind, which could then be used to alter the local concentration of a particular molecule in a specific region of the brain without actually opening the BBB. The recent clinical trials utilizing FUS and MBs to open the BBB in Alzheimer’s or glioma patients (56, 57) provide hope that other therapeutic applications of FUS and MBs, such as this one, could be introduced to the clinic in the near future. While these trials involve intentional disruption of the BBB and would thus permit large-molecule drug delivery, which our current approach does not, we hope that the sonoselective method detailed here could be used as an alternative therapy in the specific contexts where BBB opening may be contraindicated.

## Materials and Methods

### Animals.

Male C57BL/6 mice were purchased from Charles River and maintained on a 12/12 h light/dark cycle. Mice used in the experiments weighed between 22 and 28 g and were given food and water for ad libitum consumption. All animal experiments were approved by the Animal Care and Use Committee at the University of Virginia and conformed to the National Institutes of Health regulations for the use of animals in research.

### Cationic Lipid-Shelled MB Fabrication and Plasmid Conjugation.

See *SI Appendix* for details.

### MRI-Guided FUS-Mediated Plasmid Delivery.

Male C57BL/6 mice were anesthetized with an intraperitoneal (i.p.) injection of 120 mg/kg ketamine, 12 mg/kg xylazine, and 0.08 mg/kg atropine in sterilized 0.9% saline. A tail-vein catheter was inserted to permit intravenous (i.v.) injections of MBs, plasmid, and the MRI contrast agent. The heads of the mice were shaved and depilated, and the animals were then placed in a supine position over a degassed water bath coupled to an MR-compatible small animal FUS system (RK-100; FUS Instruments). The entire system was then placed in a 3-T MR scanner (Magnetom Trio; Siemens Medical Solutions). A 2-inch cylindrical transmit–receive radiofrequency (RF) coil, designed and built in-house, was placed around the mouse’s head to maximize imaging signal-to-noise ratio. Baseline T1-weighted MR images were acquired and used to select four FUS target locations in and around the right striatum.

Mice received an injection of the conjugated MBs and mCherry (for fluorescence microscopy and bulk RNA sequencing assays), mRuby (for FACS sorting and single-cell RNA sequencing assays), or luciferase (for assessing off-target transfection) plasmid (2 × 10^5^ MBs per g body weight), followed by injection of additional free plasmid to reach a total plasmid dose of 40 μg, followed by 0.1 mL of 2% heparinized saline to clear the catheter. The total plasmid dosage of 40 μg is consistent with prior studies of cationic MB-mediated gene delivery (101–103). However, since we utilize a bolus injection of MBs here (as opposed to a slow infusion), we reduced the dosage of MBs to 2 × 10^5^, which only allowed for a fraction of the plasmid to be delivered in MB-bound form. Thus, the injection of free plasmid immediately following the MBs was used to achieve the remainder of the 40-μg dose.

Sonication began immediately after clearance of the catheter. Sonications were performed at 0.1-, 0.2-, 0.3-, or 0.4-MPa PNP using a 1.1-MHz single element focused transducer (FUS Instruments) operating in 10-ms bursts, 0.5-Hz pulse repetition frequency, and 2-min total duration. These PNPs are nonderated measurements made with a hydrophone in a water tank at a target distance equivalent to the treatment distance. Immediately following the FUS treatment, mice received an i.v. injection of Gd-DPTA contrast agent (0.5 μL/g body weight; Magnevist; Bayer Health Care), and T1-weighted contrast-enhanced images were acquired to assess BBB opening. Animals were removed from the MRI and placed on a warm pad for 30 min prior to reversal of the anesthetic with antisedan (1 mg/mL). Passive cavitation analysis was performed. Details are provided in *SI Appendix*.

### Stereotactic FUS-Mediated Plasmid Delivery.

Sonications using the stereotactic frame were performed using a 1-MHz spherical-face single-element FUS transducer with a diameter of 4.5 cm (Olympus). FUS (0.1, 0.2, 0.3, or 0.4 MPa; 120 s, 10-ms bursts, 0.5-Hz burst rate) was targeted to the right striatum. The 6-dB acoustic beamwidths along the axial and transverse directions are 15 mm and 4 mm, respectively. The waveform pulsing was driven by a waveform generator (AFG310; Tektronix) and amplified using a 55-dB RF power amplifier (ENI 3100LA; Electronic Navigation Industries).

Male C57BL/6 mice were anesthetized with an i.p. injection of 120 mg/kg ketamine, 12 mg/kg xylazine, and 0.08 mg/kg atropine in sterilized 0.9% saline. A tail-vein catheter was inserted to permit i.v. injections of MBs and plasmid. The heads of the mice were shaved and depilated, and the animals were then positioned prone in a stereotactic frame (Stoelting). The mouse heads were ultrasonically coupled to the FUS transducer with ultrasound gel and degassed water and positioned such that the ultrasound focus was localized to the right striatum. Mice received an i.v. injection of the conjugated MBs and mCherry plasmid (2 × 10^5^ MBs per g body weight), followed by injection of additional free plasmid to reach a total plasmid dose of 40 μg, followed by 0.1 mL of 2% heparinized saline to clear the catheter. Sonication began immediately after clearance of the catheter. In contrast to the MR-guided experiments, which targeted three or four spots, only one location was targeted in these studies due to the increased focal region of the transducer (4 mm in the transverse direction, relative to 1 mm for the transducer in the MR-compatible system).

### Histological Processing and Immunofluorescence.

See *SI Appendix* for details.

### Cell Sorting, RNA Sequencing, and Analysis.

See *SI Appendix* for details.

### Bioluminescence Measurements.

See *SI Appendix* for details.

### Statistical Analysis.

All results are reported as mean ± SEM. “*n*” values per group are evident in all figures as all individual data points are shown. Details of statistical testing are provided in the figure legends (GraphPad Prism 7). Significance was assessed at *P* < 0.05.

### Data Availability.

Bulk (GSE141728) and single cell (GSE 141922) RNA sequencing data have been deposited in the Gene Expression Omnibus database (https://www.ncbi.nlm.nih.gov/geo). All remaining data generated or analyzed during this study are included in this paper.

## Supplementary Material

Supplementary File
