# Supplementary material for: Sonoselective transfection of cerebral vasculature without blood–brain barrier disruption
Source: Proc Natl Acad Sci U S A. 2020 Mar 2;117(11):5644–54. doi: 10.1073/pnas.1914595117 (PMC7084076; doi:10.1073/pnas.1914595117)
Supplement: Supplementary File [file pnas.1914595117.sapp.pdf]

PNAS
www.pnas.org

Supplementary Information for

Sonoselective Transfection of Cerebral Vasculature without Blood-Brain Barrier Disruption

Catherine M. Gorick[1], Alexander S. Mathew[1], William J. Garrison[1], E. Andrew Thim[1], Delaney G. Fisher[1], Caitleen A. Copeland[1], Ji Song[1], Alexander L. Klibanov[1,2], G. Wilson Miller[1,3], and Richard J. Price[1,3*]

Richard J. Price
Email: rprice@virginia.edu

This PDF file includes:

Supplementary Materials and Methods
Figures S1 to S5
Table S1
SI References

www.pnas.org/cgi/doi/10.1073/pnas.1914595117

Supplementary Information Text
Materials and Methods.

Cationic Lipid-Shelled Microbubble Fabrication

To synthesize the cationic lipid-shelled MBs, we made a mixture of 2 mg/ml 1,2-distearoyl-sn-glycero-3-phosphocholine (DSPC; Avanti Polar Lipids, Alabastar, Alabama), 2 mg/ml polyethylene glycol 6000 monostearate (PEG 6000 MS; Stepan Kessco, Northfield, Illinois), and 0.8 mg/ml 1,2-distearoyl-3-trimethylammonium-propane (DSTAP; Avanti Polar Lipids, Alabastar, Alabama) in 0.9% NaCl (Baxter, Deerfield, Illinois). The mixture was filtered through a 0.2 um Nylon sterile filter, sparged with decafluorobutane gas (F2 Chemicals Ltd; Preston, United Kingdom), and then sonicated at the highest power (20 kHz, 30 s) with an ultrasound disintegrator (XL2020; Misonix, Farmingdale, New York) to generate the MBs. MBs were aliquoted into 13 mm glass vials, which were stoppered for storage after filling the headspace with decafluorobutane gas. The MBs were cleaned by flotation centrifugation before each experiment to remove residual micelles. An aliquot of the MB solution was centrifuged at 1000 rpm for 10 minutes, and the infranatant was removed and the bubbles resuspended in degassed saline. This process was repeated three times before the final resuspension of the bubbles at a concentration between 1.5 and $2*10^9$ MBs/ml. MBs were sized and counted using a Coulter counter (Multisizer 3; Beckman Coulter, Fullerton, California).

Plasmid Preparation and Conjugation to Microbubbles

The mCherry2-C1 and mRuby2-N1 plasmids were a gift from Michael Davidson (Addgene plasmids #54563 and #54614; http://n2t.net/addgene:54563 and http://n2t.net/addgene:54614; RRID:Addgene_54563 and RRID:Addgene_54614). The luciferase plasmid was a gift from William Kaelin (Addgene plasmid #18964; http://n2t.net/addgene:18964). The plasmids feature either an mCherry2, mRuby2, or luciferase gene under the control of a CMV enhancer and promoter for constitutive expression. Our studies demonstrate that peak expression is achieved by these plasmids by 24 hours after transfection and is maintained for about 1 day before beginning to decline. The plasmids were provided from Addgene (Watertown, Massachusetts) in the form of agar stabs of DH5α *E. coli* transformed with the plasmids. The bacteria were expanded in LB media (Sigma-Aldrich, St. Louis, Missouri) containing kanamycin (Sigma-Aldrich), and then the plasmids were collected and purified using an Endo-Free Maxiprep Kit (Qiagen, Germantown, Maryland). Plasmid was resuspended in Tris-EDTA buffer at a

concentration between 400 and 500 ng/ul and stored at -20 degrees Celsius. Plasmid concentration was determined using a NanoDrop ND-1000 spectrophotometer (NanoDrop Technologies, Wilmington, Delaware).

On the morning of experiments, the appropriate purified plasmid was thawed and added to the cleaned MB solution at a ratio of 1.5 ug plasmid per 10^7 microbubbles. This ratio is consistent with prior studies of DNA binding to cationic MBs(1, 2). This mixture was allowed to incubate at room temperature for 10 min to permit the electrostatic coupling of the positively-charged bubbles and negatively-charged DNA, and was then stored on ice until use. Roughly 20-25% of the plasmid added to the MBs bound, for a total of 0.03 to 0.035 pg per MB. We did not observe significant changes to the size distributions of the MBs after conjugation to plasmid (Figure S1).

Passive Cavitation Detection

Acoustic emissions were detected with a 2.5 mm wideband unfocused hydrophone mounted in the center of the transducer. Acoustic signal was captured using a scope card (ATS460, Alazar, Pointe-Claire, Canada) and processed using an in-house built MATLAB algorithm. Acoustic emissions at the fundamental frequency, harmonics (2f, 3f, 4f), sub harmonic (0.5f), and ultra-harmonics (1.5f, 2.5f, 3.5f) were assessed by first taking the root mean square of the peak spectral amplitude (V_{rms}) in each frequency band after applying a 200 Hz bandwidth filter, and then summing the product of V_{rms} and individual sonication duration over the entire treatment period. Broadband emissions were assessed by summing the product of V_{rms} and individual sonication duration for all remaining emissions over the entire treatment period.

Histological Processing

Immediately following euthanasia via an overdose of pentobarbital sodium and phenytoin sodium, animals were perfused via the carotid arteries with 10 ml of 2% heparinized 0.9% saline followed by 5 ml of 4% paraformaldehyde. The brains were suffusion-fixed in 4% paraformaldehyde for 24 hours at 4 degrees Celsius, followed by desiccation in 30% sucrose for 24 hours at 4 degrees Celsius. The desiccated brains were then equilibrated in OCT compound for 1 hour prior to flash freezing and storage at -80 degrees Celsius. The brains were then mounted with OCT and sectioned using a cryostat (Leica, Buffalo Grove, Illinois) into 5 um thick sections.

Immunofluorescence – GLUT1 Staining of Endothelium

To assess endothelial selectivity of transfection using GLUT1 as an endothelial marker, mounted sections were washed 3× for 10 min in PBS with 0.1% Tween 20 and incubated with blocking solution (1% normal goat serum, 2% bovine serum albumin in 0.1% Tween 20 in PBS) for one hour. Sections were next incubated overnight with rabbit anti-mCherry (1:400; Abcam, Cambridge, Massachusetts) and mouse anti-GLUT1 (1:200; Abcam). After washing 3× for 10 min in PBS with 0.1% Tween 20, sections were incubated for 1.25 hours at room temp with Alexa Fluor 555-conjugated goat anti-rabbit IgG (1:500; Thermo Scientific, Waltham, Massachusetts), Alexa Fluor 488-conjugated goat anti-mouse IgG (1:200; Thermo Scientific), and Draq5 (1:1000; Thermo Scientific). After washing 3× for 10 min in PBS, sections were mounted using Prolong Diamond (Thermo Scientific).

Sections were imaged on a Nikon Eclipse TE2000 confocal microscope equipped with a 20× oil objective. Endothelial selectivity was assessed using ImageJ by manually comparing co-localization of mCherry expression with GLUT1 expression. At least three representative fields of view were counted from the FUS-treated region of the brain, as well as three fields of view from the contralateral side of the brain.

Fluorescent Histochemistry – BS-I Lectin Staining of Endothelium

To assess endothelial selectivity of transfection using BS-I lectin as an endothelial marker, mounted sections were washed 3× for 10 min in PBS with 0.1% Tween 20 and incubated with blocking solution (1% normal goat serum, 2% bovine serum albumin in 0.1% Tween 20 in PBS). Sections were next incubated overnight with rabbit anti-mCherry (1:400; Abcam). After washing 3× for 10 min in PBS with 0.1% Tween 20, sections were incubated for 1 hr at room temp with Alexa Fluor 555-conjugated goat anti-rabbit IgG (1:500; Thermo Scientific), Alexa Fluor 488-conjugated BS-I lectin (1:300; Thermo Scientific), and Draq5 (1:1000; Thermo Scientific). After washing 3× for 10 min in PBS, sections were mounted using Prolong Diamond (Thermo Scientific).

Sections were imaged on a Nikon Eclipse TE2000 confocal microscope equipped with a 20× oil objective. Endothelial selectivity was assessed using ImageJ by manually comparing co-localization of mCherry expression with GLUT1 expression. At least three representative fields of view were counted from the FUS-treated region of the brain, as well as three fields of view from the contralateral side of the brain.

Bioluminescence Measurements

To assess biodistribution of the transgene and off-target transfection, mice received MBs conjugated to a luciferase plasmid and were treated with FUS in the right hemisphere of the brain. One day later, the mice were sacrificed and their organs (brain, lungs, heart, kidneys, and liver) were harvested and placed in a solution of D-Luciferin (150 ug/ml; Gold Biotechnology, St. Louis, MO) in PBS for 5 minutes. The organs were then imaged using an IVIS100 imaging system (Xenogen, Alameda, CA). Photons were collected and integrated for a period of 1 minute. Images were then processed using Xenogen's Living Image software.

Bulk RNA Sequencing and Analysis

Immediately following euthanasia, the mouse brains were harvested and the front right quadrants (FUS-treated region) were excised, placed in RNAlater (Qiagen), and stored at -80 °C. RNA extraction was performed using the RNeasy Mini Kit (Qiagen). mRNA was isolated using the NEBNext Poly(A) mRNA Magnetic Isolation Module (New England Biolabs, Ipswich, Massachusetts) followed by library preparation using the NEBNext Ultra II Directional RNA Library Prep Kit for Illumina (New England Biolabs). Sequencing was performed using a NextSeq 500 (Illumina, San Diego, California) at a target depth of 25 million 2 x 75 bp paired end reads per sample. Reads were quasi-mapped to the mouse genome (mm10 assembly) and quantified at the transcript level using Salmon v0.11.2(3) followed by summary to the gene level using tximport v1.10.1(4). Differential gene expression was performed with DESeq2 v1.22.2(5). Gene set enrichment analysis was performed with the MSigDB canonical pathways gene sets(6) using FGSEA v1.8.0(7) run with 10,000 permutations.

Fluorescence Activated Cell Sorting (FACS)

Immediately following euthanasia, the mouse brains treated with 0 MPa, 0.1 MPa, 0.2 MPa, or 0.4 MPa (n = 3 per group) were harvested. The front right quadrants of each brain were excised and made into single cell suspensions using the Adult Brain Dissociation Kit (Miltenyi Biotech, Bergisch Gladbach, Germany). Suspensions were pooled by treatment group and incubated briefly with SYTOX Green Nucleic Acid Stain (1:500,000; Thermo Scientific) to identify live and dead cells. mRuby$^+$ cells were isolated from 0.1 MPa, 0.2 MPa, and 0.4 MPa cell suspensions using a BD Influx Cell Sorter (BD Biosciences, San Jose, California) with the 100 µm nozzle at 20 psi. The sort gate was

established using the 0 MPa cells as a reference. Live singlet $mRuby^+$ cells were collected for single cell RNA-sequencing. FACS data were analyzed using FCS Express 6 software.

Single Cell RNA Sequencing and Analysis

After FACS, 0 MPa (unsorted), 0.1 MPa ($mRuby^+$), 0.2 MPa ($mRuby^+$), and 0.4 MPa ($mRuby^+$) single cell libraries were generated using the Chromium Controller (10X Genomics, Pleasanton, CA) with the Chromium Single Cell 3' GEM, Library & Gel Bead Kit v3 (10X Genomics) and Chromium Single Cell B Chip Kit (10X Genomics). An average of 1531 cells per condition were sequenced on a NextSeq 500 (Illumina) at an average depth of 87,184 reads per cell. The CellRanger v3.0.2 pipeline was implemented to first convert bcl2 reads to FASTQ files followed by alignment to the mm10 (Ensembl 84) mouse reference genome and filtering. All further single cell analysis was performed in R using Seurat v3.0.2(8) with default parameters unless otherwise specified. Cell clusters were computed by graph-based clustering and subsequently identified by comparing the top 20 globally distinguishing markers with those having high cell-type specificity scores in the PanglaoDB webserver(9). Differential gene expression between endothelial subsets was performed using the MAST framework(10). Gene set enrichment analysis was performed with the MSigDB canonical pathways gene sets(6) using FGSEA v1.8.0(7) run with 100,000 permutations and sign(log2 fold change)*-log10(adjusted p value) as the ranking metric.

Supplemental Figures

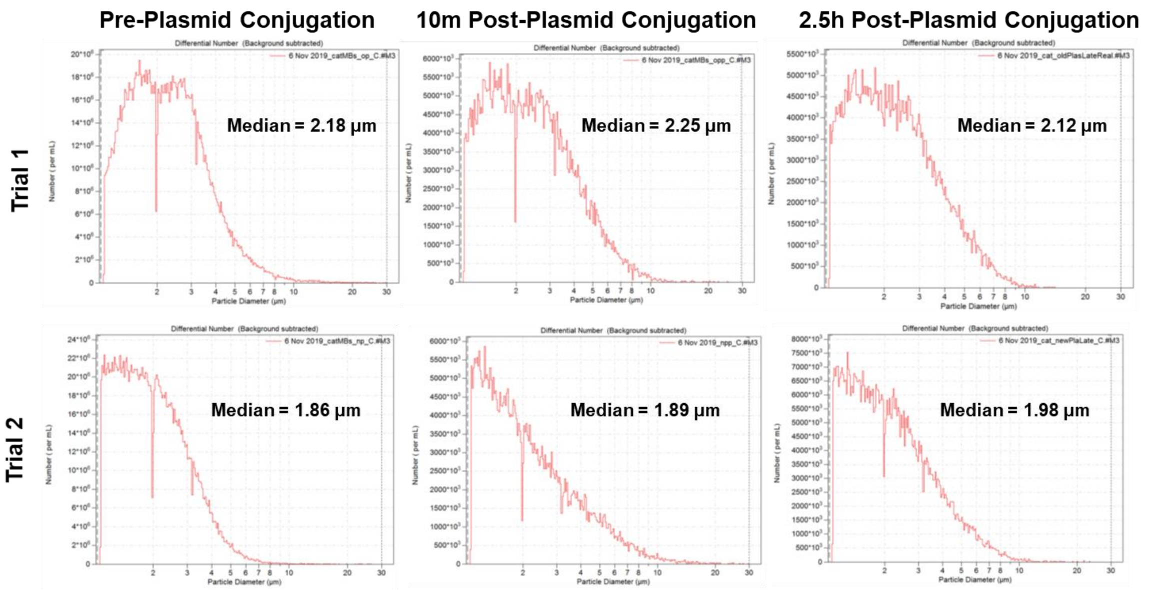

Figure S1. Size distributions of cationic MBs, pre- and post-plasmid conjugation. Data from 2 independent trials show that plasmid conjugation does not affect MB size. MB-plasmid conjugates remain stable for at least 2.5 hours. Total MB-bound plasmid was 0.034 pg/MB and 0.030 pg/MB, respectively, for Trials 1 and 2.

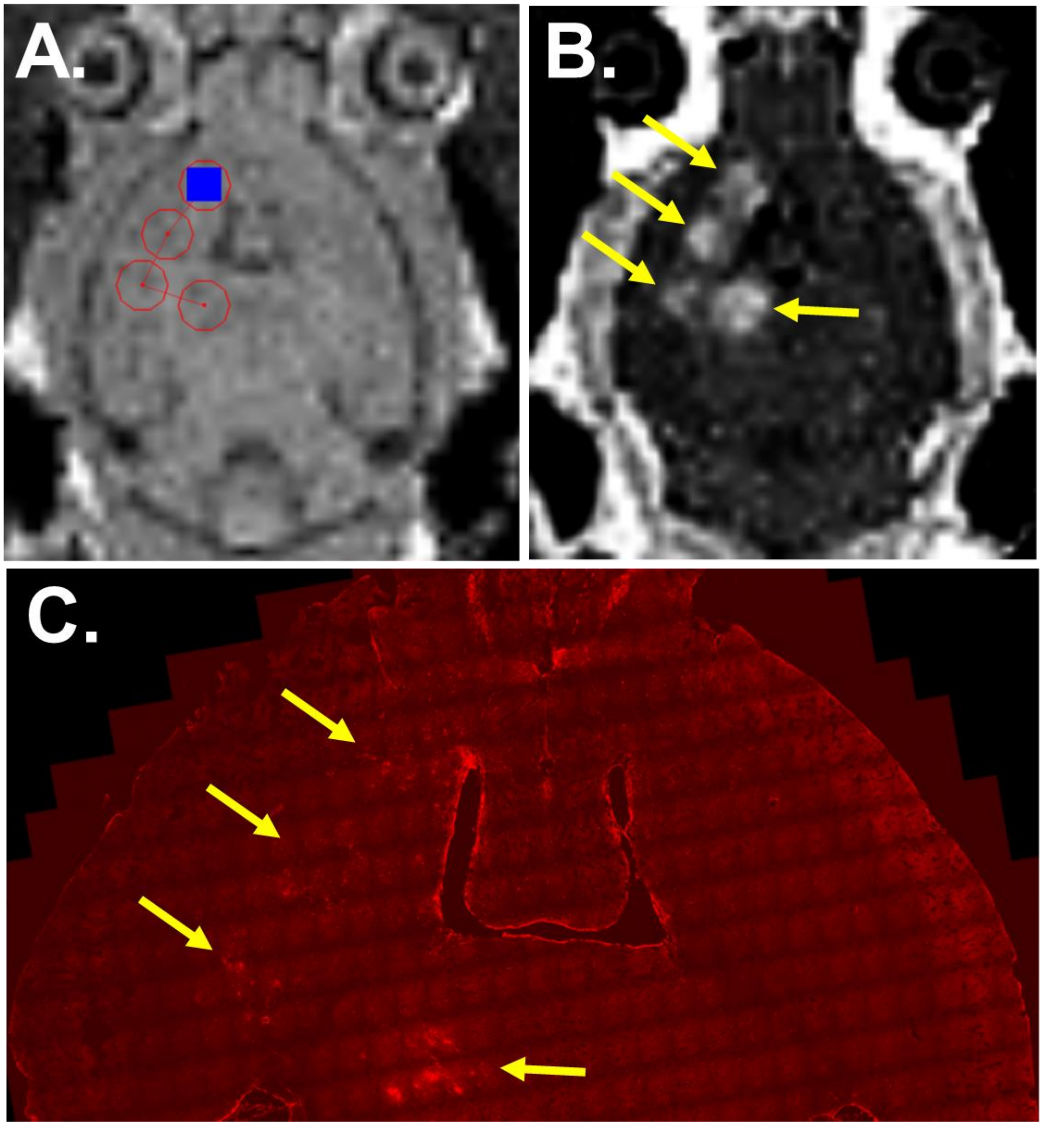

Figure S2. mCherry expression colocalizes with FUS treated brain regions. A) Pre-FUS MRI with overlaid 4-spot treatment plan (red circles). B) Post-FUS (0.4 MPa) BBB opening is evident using T1 contrast MRI, with a spatial distribution corresponding to the treatment plan. C) mCherry expression in the same brain is detectable in the FUS-treated regions (arrows).

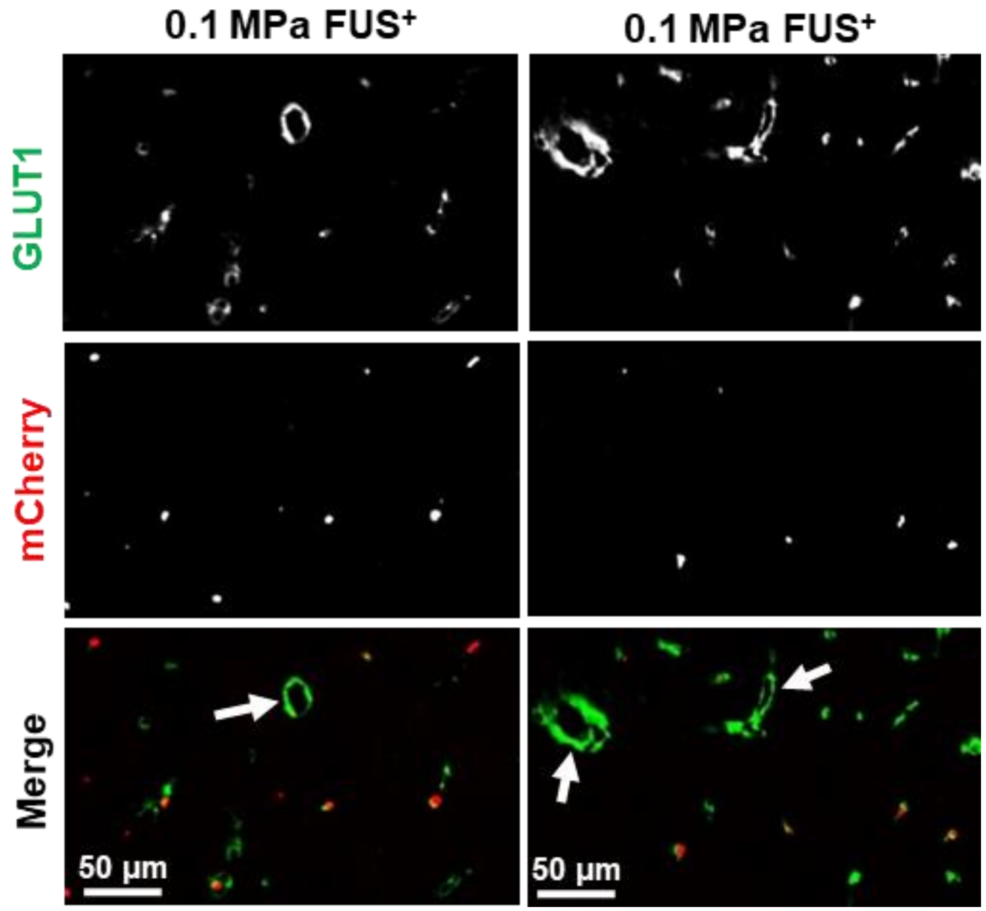

Figure S3. Sonoselective transfection of cerebrovascular endothelium using 0.1 MPa FUS is restricted to small (i.e. < 5 µm) microvessels. Representative confocal images of FUS^+ (0.1 MPa) brain tissue sections showing expression of mCherry reporter gene (red) with respect to endothelial cells (GLUT1; green). Yellow arrowheads denote co-localization of mCherry with endothelium in capillary-sized microvessels. White arrows denote larger microvessels, all of which are devoid of mCherry expression.

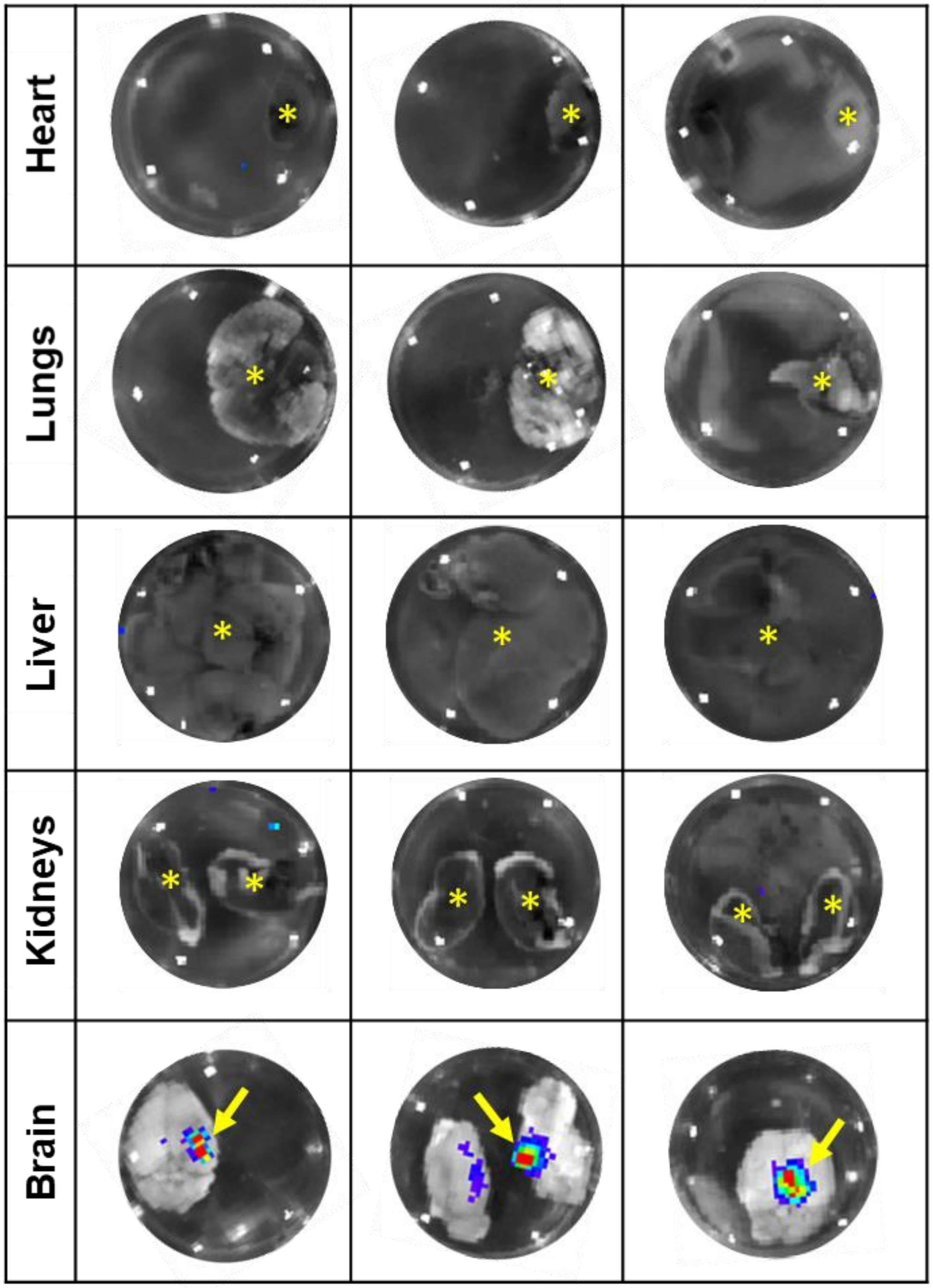

Figure S4. Luciferase transgene was detected in 0.1 MPa FUS-treated brains, but not in off-target organs. Bioluminescence images of heart, lungs, liver, kidneys, and brains from mice (n=3) taken 24h after luciferase plasmid was coupled to cationic microbubbles, injected I.V., and delivered to cerebrovascular endothelium under stereotactic guidance using 1 MHz FUS with a PNP of 0.1 MPa. Luciferase was robustly expressed in FUS-treated brains (arrows), but was undetectable in off-target organs (asterisks). The brain in the middle row was inadvertently bisected during processing.

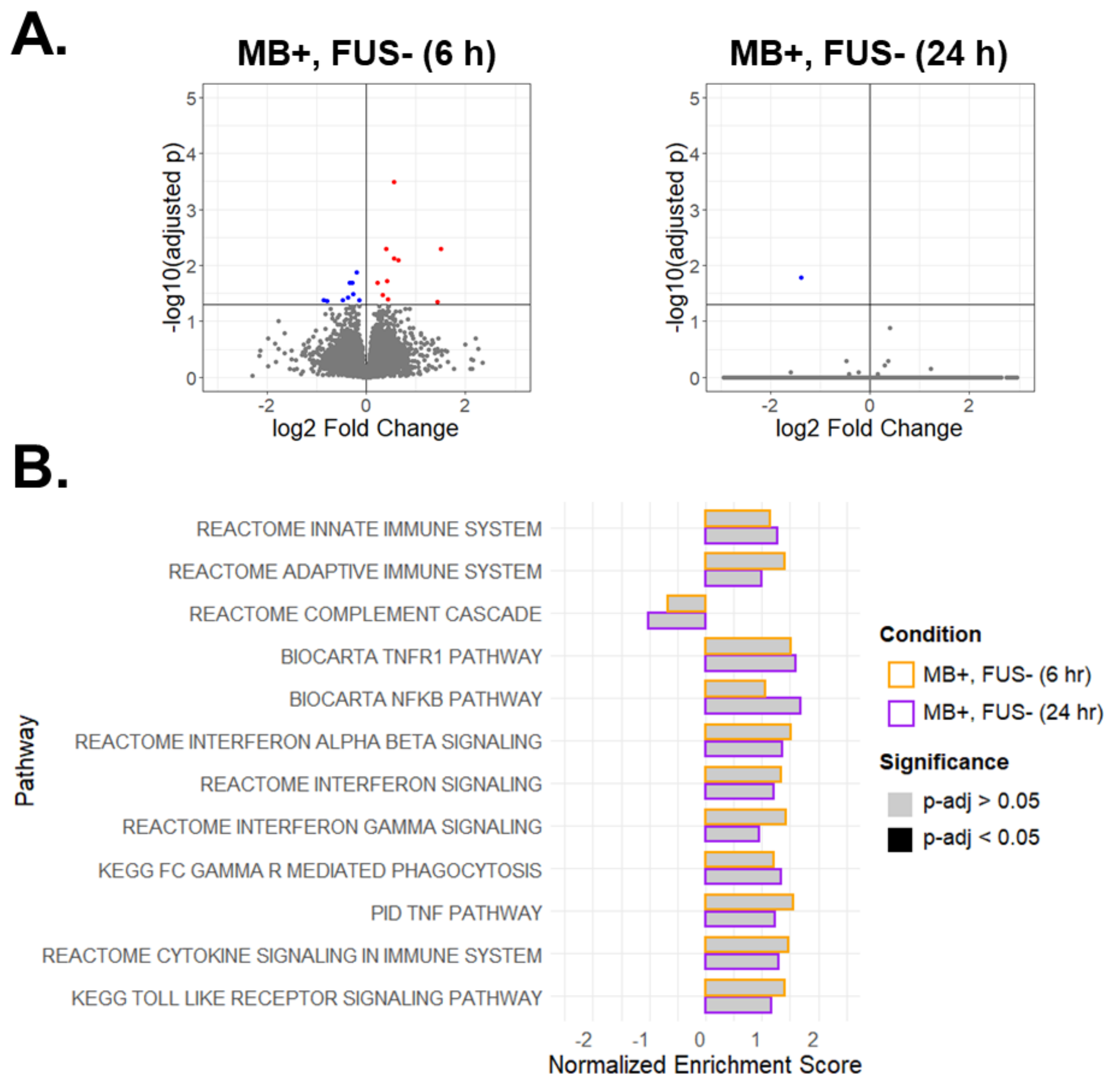

Figure S5. Systemic injection of MBs without FUS application elicits minimal transcriptomic effects in the brain. A) Volcano plots showing that, in the complete absence of FUS, systemic administration of plasmid-conjugated MBs elicits almost no differential gene expression at both 6 h and 24 h. **B)** Gene set enrichment analysis of several pathways associated with inflammation and/or other immunological responses. None of these pathways were enriched or suppressed with MB injection alone. Bar borders correspond to time post-injection and bar fills correspond to the significance of the pathway’s Normalized Enrichment Score.

	0.1 MPa		0.2 MPa		0.4 MPa	
	6 h	24 h	6 h	24 h	6 h	24 h
**IL-10**	1.0000	1.0000	1.0000	1.0000	1.0000	1.0000
**IL-11**	0.8320	1.0000	0.9998	1.0000	0.8024	0.9286
**IL-13**	1.0000	1.0000	0.9998	1.0000	1.0000	1.0000
**IL-1RA**	0.9894	1.0000	0.9998	0.9302	0.1045	0.5680
**IL-4**	0.9962	1.0000	0.9998	0.8803	0.9733	0.9765
**IL-6**	1.0000	1.0000	0.9998	1.0000	0.9551	0.9037

Table S1. FDR adjusted p-values for relative expression levels of "anti-inflammatory" transcripts. Transcript expression significance levels are shown for FUS+MB groups at all 3 tested peak-negative pressures in comparison to the "MB only" control group. No significant differences were observed.

SI References

1. Carson AR, et al. (2011) Gene therapy of carcinoma using ultrasound-targeted microbubble destruction. *Ultrasound Med Biol* 37(3):393–402.
2. Christiansen JP, French BA, Klibanov AL, Kaul S, Lindner JR (2003) Targeted tissue transfection with ultrasound destruction of plasmid-bearing cationic microbubbles. *Ultrasound Med Biol* 29(12):1759–67.
3. Patro R, Duggal G, Love MI, Irizarry RA, Kingsford C (2017) Salmon provides fast and bias-aware quantification of transcript expression. *Nat Methods* 14(4):417–419.
4. Soneson C, Love MI, Robinson MD (2016) Differential analyses for RNA-seq: transcript-level estimates improve gene-level inferences. *F1000Research* 4:1521.
5. Love MI, Huber W, Anders S (2014) Moderated estimation of fold change and dispersion for RNA-seq data with DESeq2. *Genome Biol* 15(12):550.
6. Liberzon A, et al. (2011) Molecular signatures database (MSigDB) 3.0. *Bioinformatics* 27(12):1739–1740.
7. Sergushichev AA (2016) An algorithm for fast preranked gene set enrichment analysis using cumulative statistic calculation. *bioRxiv*:60012.
8. Butler A, Hoffman P, Smibert P, Papalexi E, Satija R (2018) Integrating single-cell transcriptomic data across different conditions, technologies, and species. *Nat Biotechnol* 36(5):411–420.
9. Franzén O, Gan L-M, Björkegren JLM (2019) PanglaoDB: a web server for exploration of mouse and human single-cell RNA sequencing data. *Database* 2019. doi:10.1093/database/baz046.
10. Finak G, et al. (2015) MAST: a flexible statistical framework for assessing transcriptional changes and characterizing heterogeneity in single-cell RNA sequencing data. *Genome Biol* 16(1):278.
